# One-Pot Green Synthesis of Ag-Decorated SnO_2_ Microsphere: an Efficient and Reusable Catalyst for Reduction of 4-Nitrophenol

**DOI:** 10.1186/s11671-017-2204-8

**Published:** 2017-06-30

**Authors:** Min Hu, Zhenwei Zhang, Chenkun Luo, Xiuqing Qiao

**Affiliations:** College of Materials and Chemical Engineering, Hubei Provincial Collaborative Innovation Center for New Energy Microgrid, Key Laboratory of Inorganic Nonmetallic Crystalline and Energy Conversion Materials, China Three Gorges University, Yichang, 443002 Hubei People’s Republic of China

**Keywords:** Ag-decorated SnO_2_, Microsphere, Hydrothermal, Catalytic, 4-Nitrophenol, Hierarchical

## Abstract

In this paper, hierarchical Ag-decorated SnO_2_ microspheres were synthesized by a facile one-pot hydrothermal method. The resulting composites were characterized by XRD, SEM, TEM, XPS, BET, and FTIR analysis. The catalytic performances of the samples were evaluated with the reduction of 4-nitrophenol to 4-aminophenol by potassium borohydride (KBH_4_) as a model reaction. Time-dependent experiments indicated that the hierarchical microspheres assembled from SnO_2_ and Ag nanoparticles can be formed when the react time is less than 10 h. With the increase of hydrothermal time, SnO_2_ nanoparticles will self-assemble into SnO_2_ nanosheets and Ag nanoparticles decorated SnO_2_ nanosheets were obtained. When evaluated as catalyst, the obtained Ag-decorated SnO_2_ microsphere prepared for 36 h exhibited excellent catalytic performance with normalized rate constant (*κ*_nor_) of 6.20 min^−1^g^−1^L, which is much better than that of some previous reported catalysts. Moreover, this Ag-decorated SnO_2_ microsphere demonstrates good reusability after the first five cycles. In addition, we speculate the formation mechanism of the hierarchical Ag-decorated SnO_2_ microsphere and discussed the possible origin of the excellent catalytic activity.

**Electronic supplementary material:**

The online version of this article (doi:10.1186/s11671-017-2204-8) contains supplementary material, which is available to authorized users.

## Background

SnO_2_ is an important n-type semiconductor with large bandgap (Eg = 3.6 eV, at 300 K), high electron mobility, and low cost, which enable it with outstanding properties in gas sensing [1], lithium ion batteries [2], optoelectronic devices, and dye sensitized solar cells [3–8]. In the past two decades, the robust SnO_2_ material has garnered considerable attention and various nanostructures have been reported [9, 10]. Among which, three-dimensional (3D) hierarchical structures self-assembled by nanosheets building blocks are much more interesting due to their special structure and fascinating properties [11, 12]. Nevertheless, there are only a few reports on the catalytic performance of SnO_2_ and the catalytic efficiency is relatively low [13–15]. It is thus important to synthesize hierarchical SnO_2_ structures and study the catalytic performance. Especially, as we know, noble metal nanoparticles (NPs) such as Au-, Ag-, Pt-, and Pd-modified 3D hierarchical structures will show much enhanced catalytic performance [16]. However, most of the processes of the syntheses of the above noble metal-modified oxides are more complicated multi-step process and usually toxic and harmful environmentally [17]. So developing facile and efficient methods to fabricate noble metal NP-modified hierarchical SnO_2_ and studying the catalytic performance are highly desirable.

Increased contamination of our limited water resources owing to the widespread dispersion of various industrial dyes, heavy metal ions, and other aromatic pollutants are endangering our planet [18]. The 4-nitrophenol (4-NP), a well-known toxic pollutant, is widely present in industrial effluents and agriculture wastewater [19]. Among various treatment techniques, such as membrane filtration [20], photo degradation [21], adsorption [22], and chemical reduction [23–30], the chemical reduction of 4-NP to 4-aminophenol (4-AP) is a favorable route, owing to the product (4-AP) which is an important intermediate for the manufacture of analgesic and antipyretic drugs, photographic developer, corrosion inhibitor, anticorrosion lubricant, and hair-dyeing agent [31, 32]. Hence, the reduction of 4-NP to 4-AP possesses great significance for the pollution abatement and resource regeneration [33].

In this paper, we reported a green synthesis of noble metal Ag nanoparticle (NP)-modified SnO_2_ hierarchical architectures by a simple one-pot hydrothermal route without the assistant of any templates and surfactants at mild temperature. The effects of reaction time on morphologies of Ag-decorated SnO_2_ microsphere were investigated, and a possible growth mechanism of Ag-decorated SnO_2_ hierarchical structures was proposed. The catalytic results indicate the as-synthesized products exhibit excellent catalytic performance for the reduction of 4-NP to 4-AP, with normalized rate constant (*κ*_nor_) of 6.20 min^−1^g^−1^L. In addition, the Ag-decorated SnO_2_ hierarchical structures sustain high catalytic efficiency in ten cycles and show stability after the first five cycles. This obtained Ag-decorated SnO_2_ hierarchical structures may have potential applications of water contaminant treatment, and this simple one-step hydrothermal route could be extended to design other noble metal NP-modified composite with a wide range of practical applications for the future.

## Methods

### Materials

Silver nitrate (AgNO_3_, 99.8%), urea (CO(NH_2_)_2_, 99%), ammonia solution (NH_3_·H_2_O, 25~28%), and potassium borohydride (KBH_4_, 97%) were purchased from Sinopharm Chemical Reagent Co. Ltd. Sodium stannate rehydrate (Na_2_SnO_3_·3H_2_O, 98%) and 4-nitrophenol(C_6_H_5_NO_3_, 98%) were supplied by Aladdin Reagent Co. Ltd. All the materials were used without further purification.

### Synthesis of Ag-Decorated SnO_2_ Microsphere

Ag-decorated SnO_2_ powder (mole ratio of Ag:SnO_2_ = 1:1) was synthesized by one-pot hydrothermal method. In a typical procedure, 2.67 g of sodium stannate rehydrates and 0.2 g of urea were dissolved in 25 mL of ultra-pure water and stirred vigorously for 30 min to form a mixture. Then, 1.69 g of silver nitrate was dispersed in 25 mL of ultra-pure water, and then, 2.4 mL ammonium hydroxide was added into the silver nitrate solution to form silver–ammonia solution. After stirring for 5 min, the freshly prepared silver–ammonia solution was added into the mixture under magnetic stirring for 1 h. Subsequently, the resulting mixture was migrated into a 50-mL Teflon-lined autoclave and heated at 150 °C for 5, 10, 24, and 36 h. After the hydrothermal procedure, the autoclave was cooled down naturally to room temperature and the SnO_2_/Ag product was collected by centrifugation, followed by rinsing with deionized water and ethanol and drying in a vacuum oven at 60 °C. SnO_2_/Ag microsphere with different mole ratios (1.5:1, 1:1, 0.5:1, 0.01:1) of Ag to SnO_2_ are synthesized in a similar way except for the amounts of AgNO_3_ and NH_3_·H_2_O. For comparison, pure SnO_2_ and Ag were also synthesized by the similar procedure without the addition of AgNO_3_ and Na_2_SnO_3_.

### Sample Characterizations

The crystalline phase of the as-prepared samples were investigated by X-ray powder diffraction (XRD, Cu Kα radiation (*λ* = 1.5418 Å)). The scanning electron microscopy (SEM) measurements were performed on a SU-70 field emission SEM microscope with an acceleration voltage of 20 kV. Transmission electron micrograph (TEM) and high-resolution transmission electron microscopy (HRTEM) were taken on a Tecnai G2 F20 S-TWIN transmission electron microscope with an accelerating voltage of 200 kV. X-ray photo-electron spectroscopy (XPS) was performed to identify surface chemical composition and chemical states of the catalysts on a MARK II X-ray photoelectron spectrometer using Mg Kα radiation. The specific surface area of sample was evaluated by the Langmuir model and Brunauer–Emmett–Teller (BET) model based on the nitrogen adsorption isotherm obtained with a V-sorb X2008 series, while the pore size distribution was estimated by Barrett–Joyner–Halenda (BJH) theory.

### Catalytic Activity of Ag-Decorated SnO_2_ Microsphere

The reduction of 4-NP with KBH_4_ solution was used as a model reaction to study the catalytic activity of Ag-decorated SnO_2_ composites. The catalytic reduction process was carried out in a standard quartz cell with a 1-cm path length and about 4 mL volume with 0.3 mL of freshly prepared aqueous solutions of 4-NP (20 mg/L) and KBH_4_ (1.5 mg). The high molar ratio of KBH_4_ to 4-NP ensured an excess amount of the former, and hence, its concentration remained essentially constant during the reduction reaction. Upon the addition of KBH_4_ into the 4-NP solution, its color changed immediately from light yellow to dark yellow due to the formation of 4-nitrophenolate ion (formed from the high alkalinity of KBH_4_). Later, the dark yellow color faded with time (due to the conversion of 4-NP to 4-AP) after the addition of 1.5 mg of Ag-decorated SnO_2_ hybrids. The UV–Vis absorption spectra were recorded by an UV–Vis spectrometer in a scanning range of 250–500 nm at room temperature at time interval of 1 min. Several consecutive reaction rounds were measured to determine the stability of the catalyst.

## Results and Discussion

### Characterization of Ag-Decorated SnO_2_ Microsphere

**Fig. 1:**
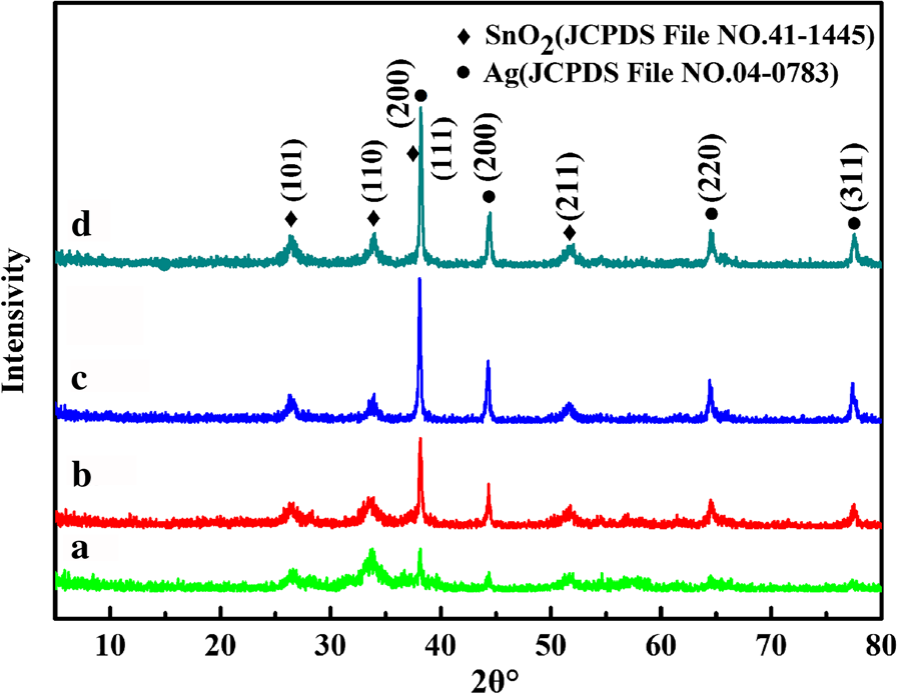
XRD patterns of the Ag-decorated SnO_2_ microspheres prepared at 150 °C for different times (*a*) 5 h, (*b*) 10 h, (*c*) 24 h, and (*d*) 36 h

The composition and phase structure of the synthesized Ag-decorated SnO_2_ powders for different times were investigated by XRD, and the corresponding patterns are shown in Fig. 1. It can be seen that the characteristic diffraction peaks match well with the tetragonal rutile phase SnO_2_ (JCPDS file no. 41-1445, *a* = 4.738Å and *c* = 3.187 Å) and face centered cubic (fcc) phase Ag (JCPDS file no. 04-0783). No diffraction peaks from any other impurities were detected indicating that the powders are the mixture of pure SnO_2_ and Ag. For the sample reacted for 5 h, the characteristic diffraction peaks at 38.12° and 44.2°, corresponding to the (111) and (200) planes of Ag, are relatively weak. With increasing hydrothermal time, the peak intensities of Ag increase and the full widths of diffraction peak decrease as well, indicating the enhanced crystallinity of Ag nanoparticles or the increased weight of Ag. This can be further verified by the XRD patterns of the samples obtained under different temperatures and different mole ratios of Ag and SnO_2_ (Additional file 1: Figure S1).

**Fig. 2:**
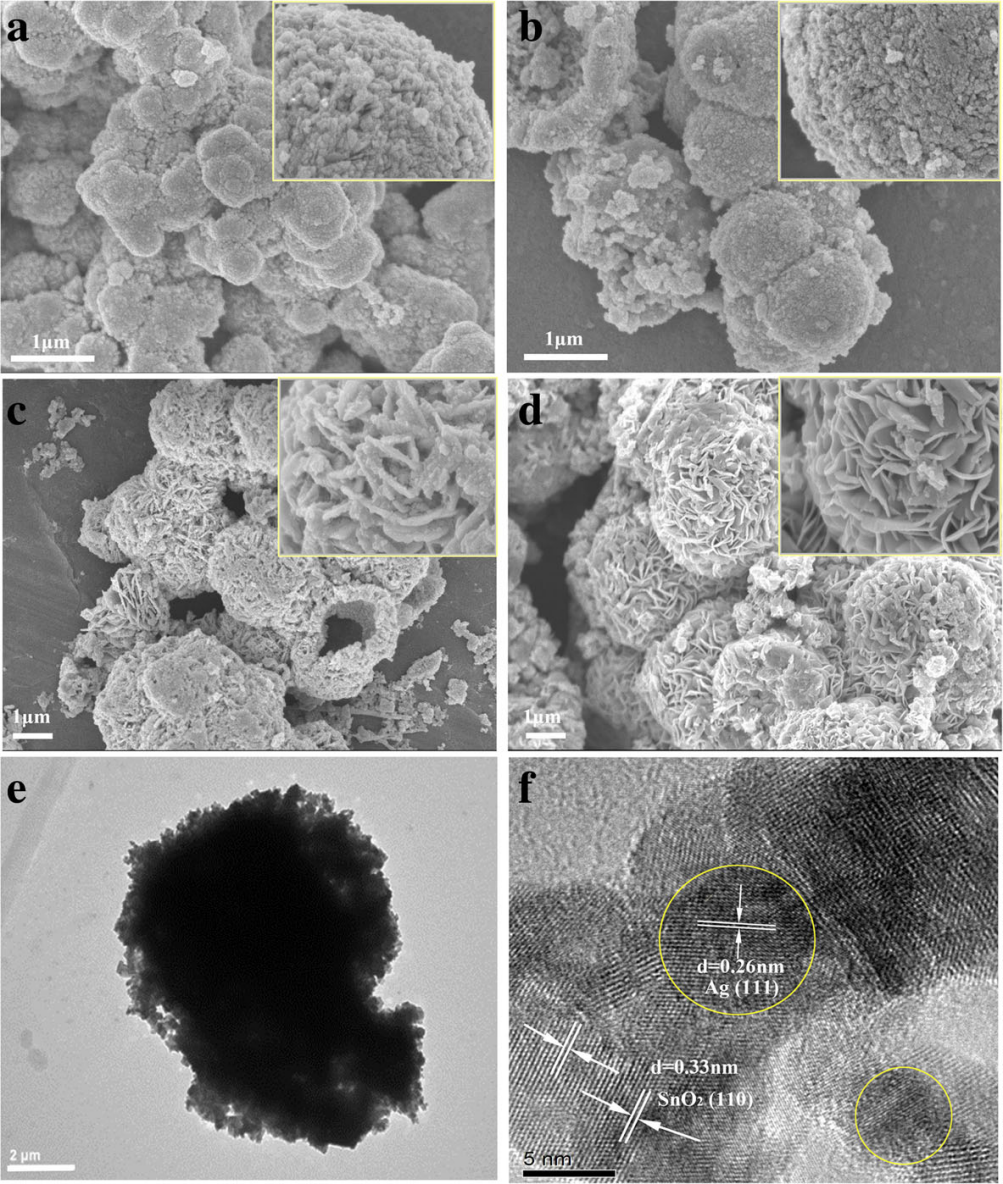
Representative FESEM images and TEM images of the Ag-decorated SnO_2_ microspheres prepared at 150 °C for different hydrothermal times **a** 5h, **b** 10h, **c** 24h, and **d** 36h and **e**, **f** low-magnification TEM image and high-resolution TEM (HRTEM) of the sample prepared for 36 h

**Fig. 3:**
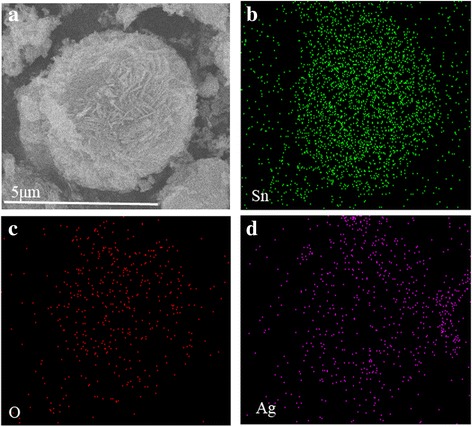
EDS element mapping of SnO_2_/Ag microspheres. **a** SEM image and element maps of **b** Sn, **c** O, and **d** Ag

The SEM images in Fig. 2 show the interesting morphological evolution of samples prepared at different hydrothermal times from 5 to 36 h. Sample prepared for 5 h was irregular microsphere, and the enlarged view of the surface of microspheres in the inset illustrating the microsphere is assembled by nanoparticles (Fig. 2a). With increasing hydrothermal time, the microsphere became more regular. Upon the hydrothermal time increased to 24 h (Fig. 2c), the microsphere grew larger at the expense of the smaller nanoparticles and the surface nanoparticles self-assembled into nanosheets. These nanosheets assembled to form a hierarchical microsphere structure. When further increasing the hydrothermal time to 36 h, the coarse nanosheets became smoothed and the microspheres with diameters ranging from 2 to 4 μm are more uniform. Further increase of the hydrothermal time led no obvious change of the morphology and crystalline (not shown in this paper). The morphology of the sample prepared for 36 h was further observed via TEM and HRTEM. As shown in Fig. 2e, the obtained SnO_2_/Ag is of microsphere morphology with diameter of ~5 μm and the microsphere is assembled from nanosheets. In the typical HRTEM image (Fig. 2f), Ag NPs with an average size of about 5 nm were formed and homogeneously distributed to SnO_2_. The lattice fringes of *d* = 0.26 nm spacing can be assigned to the Ag (111) planes while the lattice fringes of *d* = 0.33 nm can be assigned to the (110) plane of SnO_2_, respectively. To further illustrate the uniform distributions of Ag nanoparticles in the microsphere, element mapping analysis of the SnO_2_/Ag microsphere was performed (Fig. 3). As shown in the Fig. 3, the map of Ag, Sn, and O elements are fit into the sample morphology, indicating that Ag nanoparticles are uniformly dispersed in the microspheres.

**Fig. 4:**
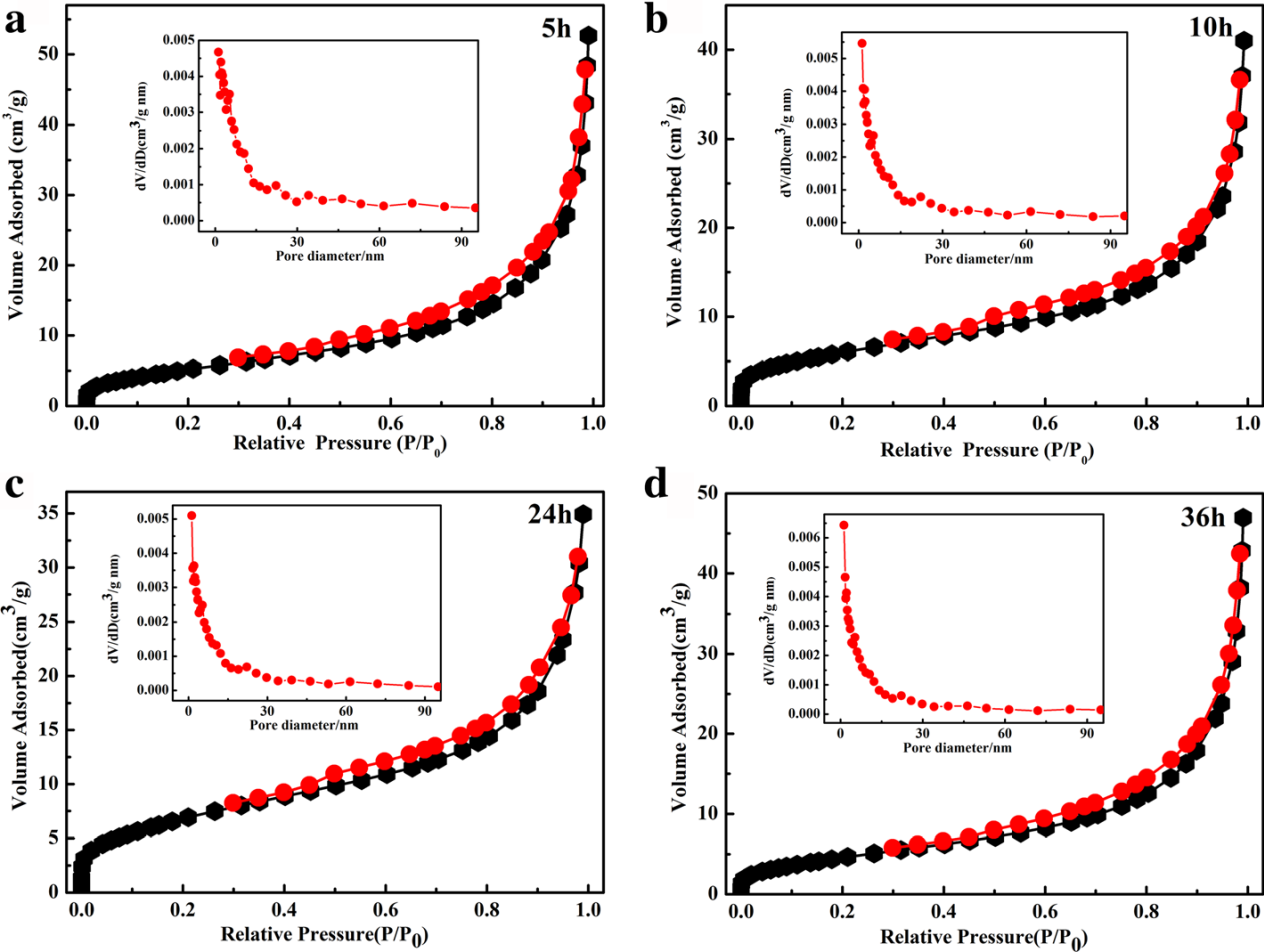
Typical nitrogen adsorption–desorption isotherm of the prepared SnO_2_/Ag microspheres prepared at 150 °C for different hydrothermal times **a** 5 h, **b** 10 h, **c** 24 h, and **d** 36 h

The N_2_ adsorption–desorption isotherms of samples and their corresponding pore size distribution are illustrated in Fig. 4. All of the samples exhibited type IV isotherms with H_3_ hysteresis loop, signifying typical mesoporous structures of uniform pore size [34]. The BET-specific surface areas were determined as 21.8, 22.4, 24.6. and 25.7 m^2^ g^−1^, respectively. Inset depicts the pore size distributions of samples. The pore size distribution is mono-modal for all the samples. The average pore diameter is ~2 nm for the as-hierarchical Ag-decorated SnO_2_ powders. It is noted that the calculated BET surface area and mean pore diameter has no obvious change with increasing hydrothermal time.

**Fig. 5:**
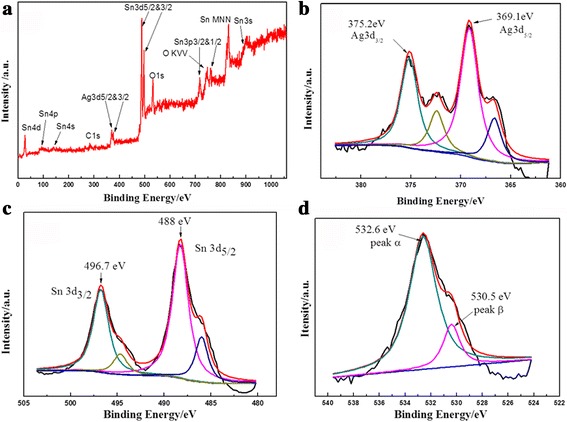
Representative XPS spectra of SnO_2_/Ag microspheres prepared at 150 °C for 36 h. **a** XPS full spectra. High-resolution spectra of the elements **b** Ag, **c** Sn, and **d** O

XPS was used to examine the chemical states and surface composition of Ag-decorated SnO_2_ microspheres. Wide survey scans were recorded first followed by a detailed scanning of the edges of each element such as Sn 3d, Ag 3d, and O 1s (Fig. 5). It may be mentioned that the charging effect on the sample was corrected by setting the binding energy of the carbon (C 1s) at 284.6 eV and this carbon peak was used as a reference position for scaling all other peaks. As shown in Fig. 5b, the peak appears as a spin–orbit doublet at 369.1 eV (Ag 3d_5/2_) and 375.2 eV (Ag 3d_3/2_) for Ag^0^ [35, 36] in the product. The two satellite peaks at 366.5 and 372.3 eV can be account for Ag 3d in Ag-decorated SnO_2_ nanocomposites [37]. Furthermore, two XPS peaks located at 488 and 496.7 eV are relevant to Sn 3d_5/2_ and Sn 3d_3/2_, indicating the presence of Sn^4+^ in SnO_2_. And the peaks around 485.7 and 494.7 eV may be caused by the binding between Sn and Ag [38, 39]. The slightly binding energy shift of these elements in Ag-decorated SnO_2_ microsphere means electrons may transfer between Ag and SnO_2_, demonstrating strong interaction between Ag nanoparticles and SnO_2_ nanosheets rather than simply physical contact. The strong interaction is advantageous for the electron transfer among the adjacent particles, which can improve the catalytic activities and be beneficial to some similar phenomenon, which was observed in other literatures [38–40]. In Fig. 5d, O 1s spectra at 530.5 eV corresponded to the lattice oxygen while the peak at 532.6 eV corresponds to chemisorbed oxygen or hydroxyl ions such as O^−^, O_2_^−^, or OH^−^ at the surface of SnO_2_ [41–44].

### Catalytic Reduction of 4-NP

**Fig. 6:**
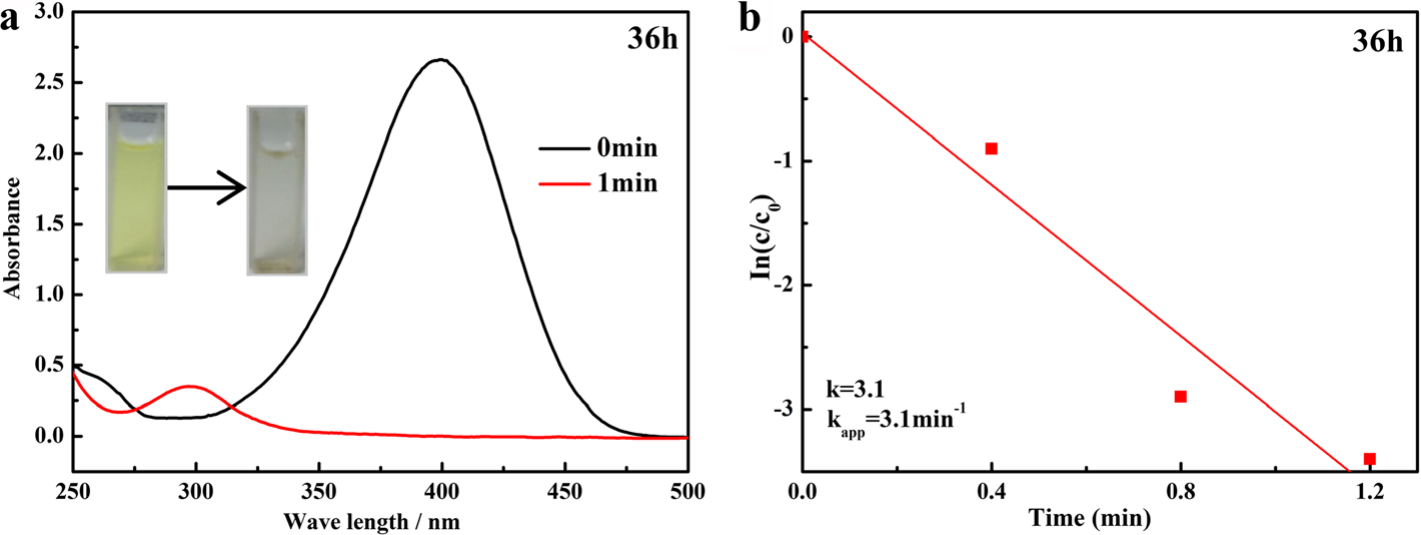
**a**, **b** Time-dependent UV–Vis absorption spectra and the plot of ln(*C*_*t*_/*C*_0_) versus reaction time for the reduction of 4-NP of the sample prepared at 150 °C for 36 h

**Fig. 7:**
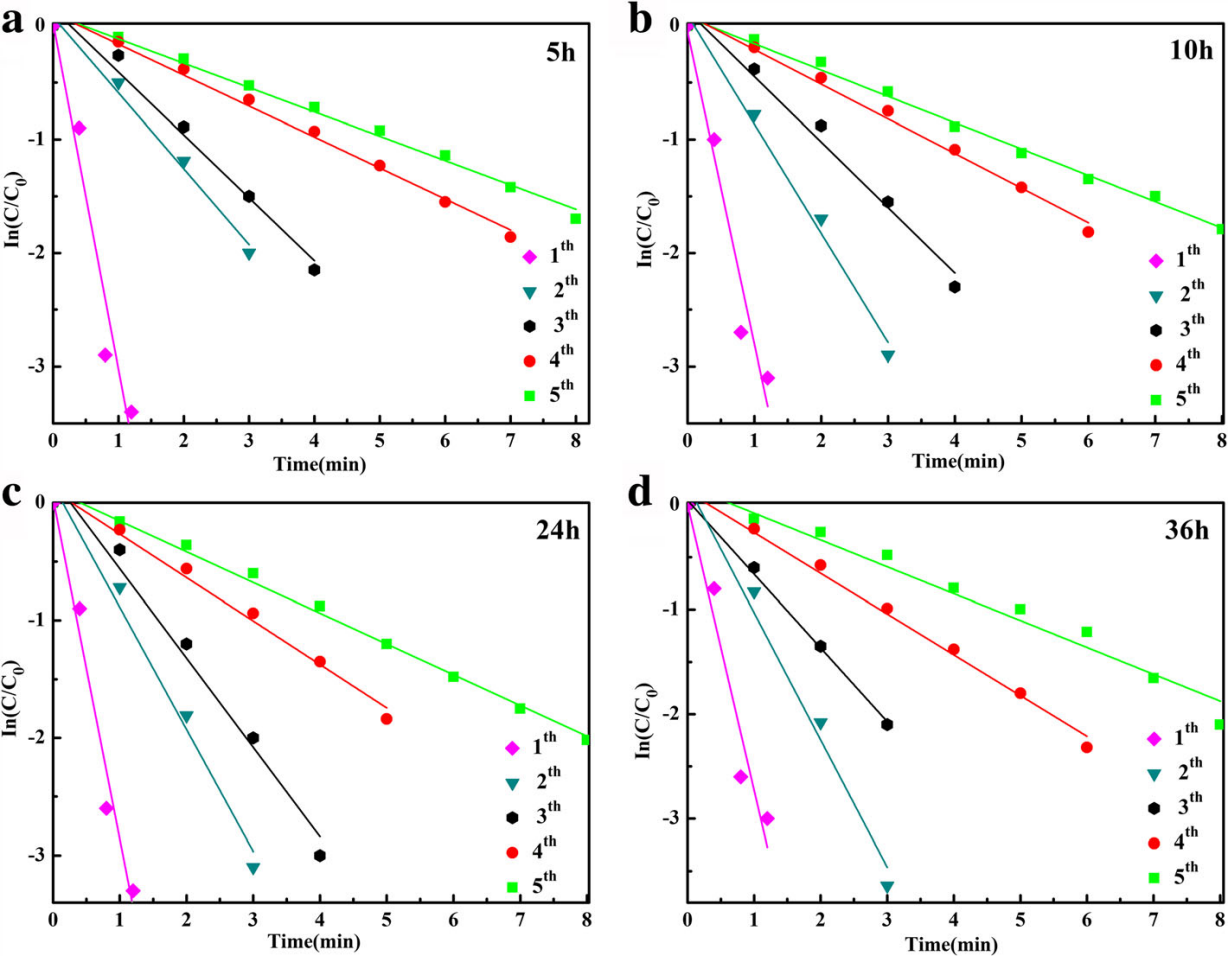
Plot of ln(*C*_*t*_/*C*_0_) versus reaction time in the presence of Ag-decorated SnO_2_ microspheres prepared for different hydrothermal times **a** 5 h, **b** 10 h, **c** 24 h, and **d** 36 h

The reduction of 4-NP by KBH_4_ in the presence of catalyst is a well-studied green chemical reaction and was chosen as the model reaction to study the catalytic activity of the as-prepared Ag-decorated SnO_2_ composites. The UV–Vis absorption spectrum with a maximum absorption at 400 nm is formed due to the nitro compound. With the Ag-decorated SnO_2_ catalyst added, the absorption peak at 400 nm, ascribed to nitro compounds, decreased sharply in 1 min and a new peak at 300 nm corresponding to 4-AP appeared, indicating the catalytic reduction of 4-NP had proceeded successfully (Fig. 6a). Considering of the excess KBH_4_, its concentration can be assumed to be a constant during the reaction. Therefore, a pseudo first-order kinetic equation can be applied to evaluate the catalytic rate. The kinetic equation of the reduction can be written as follows:

1 $$ \frac{dC}{dt}={\kappa}_{\mathrm{app}}{C}_t\; or\;\mathrm{In}\left(\frac{C_t}{C_0}\right)=\mathrm{In}\left(\frac{A_t}{A_0}\right)=-{\kappa}_{\mathrm{app}} t $$

where the ratios of 4-NP concentrations *C*_*t*_ (at time *t*) to its initial value *C*_0_ (*t* = 0) were directly given by the relative intensity of the respective absorbance A_t_/A_0_, *κ*_app_ corresponds to the apparent rate constant. The apparent rate constant, *κ*_app_, was calculated as 3.10 min^−1^ for the reduction of 4-NP of the prepared Ag-decorated SnO_2_ microsphere at 150 °C for 36 h (Fig. 6b). In order to further assess the catalytic performance of the Ag-decorated SnO_2_, all the samples prepared for different hydrothermal time were carried out to catalytic reduction of the 4-NP. The UV–Vis absorption spectra of the reduction are shown in Additional file 1: Figure S2–S5, and the corresponding plots of ln(*C*_*t*_/*C*_0_) versus time are shown in Fig. 7. It is clear that almost 100% of 4-NP can be reduced within 1 min of the first cycle. With the increase of cycle times, the time is longer. Nevertheless, over 80% of 4-NP can be reused within 8 min. It can be observed that ln(*C*_*t*_/*C*_0_) values show good linear correlation with the reaction time for all catalysts, indicating that the reduction follows a first-order reaction law. The calculated apparent rate constants *κ*_app_ of different cycles for all samples are shown in Table 1.

**Table 1:** The apparent rate constants *κ*_app_ of different cycles for all samples

Catalyst	*κ*_app_ (min^−1^)				
Sample (h)	1st	2nd	3rd	4th	5th
5	2.94	0.68	0.59	0.3	0.24
10	3.00	0.96	0.6	0.32	0.28
24	3.04	1.10	0.76	0.38	0.3
36	3.10	1.24	0.76	0.43	0.32

**Fig. 8:**
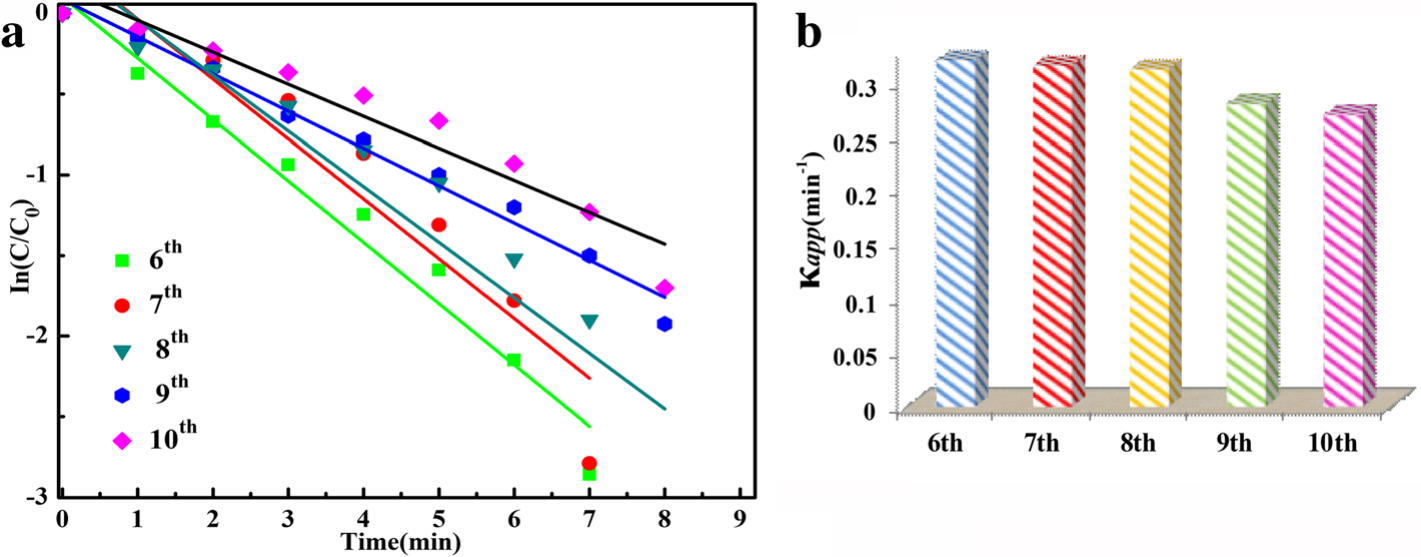
**a**, **b** Plot of ln(*C*_*t*_/*C*_0_) versus reaction time of the sixth to tenth cycles for the 36 h sample

As shown in Fig. 7 and Table 1, the apparent rate constants (*κ*_app_) increase with the extension of the hydrothermal time and decrease with the cycle times, especially for the first and second cycles. The decreases of rate constant may due to the peeling off and coagulation of Ag NPs from the microsphere during the centrifugation. In order to prove the stability of the sample prepared in the work, the separated catalyst (prepared for 36 h) was reused to catalytic reduction of 4-NP for more than five cycles. The time-dependent UV–Vis absorption spectra of the sixth cycle to tenth cycle are shown in Additional file 1: Figure S6. The corresponding apparent rate constants (*κ*_app_) shown in Fig. 8 show there is only a slight decrease in the *κ*_app_ value with the increasing of successive cycles, indicating that after the first five cycles, the catalysts are much more stable than the freshly prepared samples. This proves that the as-prepared Ag-decorated SnO_2_ samples possesses good stability for the catalytic reduction of 4-NP to p-AP by KBH_4_ and can be used as an alternative active and stable catalyst for the catalytic reduction of 4-NP.

Also, the FTIR spectra of the catalyst before and after five cycles and ten cycles of catalytic reduction were shown in ESI. As shown in Additional file 1: Figure S7, after five and ten cycles of catalytic reduction, the main peaks of the samples were almost the same with the as-prepared sample and this illustrates that the catalysts are very stable.

In order to compare our results with other catalysts in the literature, we evaluated the catalytic ability of Ag-decorated SnO_2_ by normalizing the *κ*_app_ values to *κ*_nor_ [45, 46]. The normalized rate constant *κ*_nor_ (*κ*_nor_ = *κ*_app_/*c*_cat_, where *c*_cat_ is the concentration of the catalyst) is a key indicator for estimating catalytic activity. The normalized rate constants *κ*_nor_ were calculated to be 6.20, 0.64, and 0.54 min^−1^g^−1^L of the first cycle, fifth cycle, and tenth cycle for the SnO_2_/Ag microsphere reacted for 36 h, respectively. The comparison of *κ*_nor_ of the SnO_2_/Ag (36 h) and other catalysts in literature are shown in Table 2. From Table 2, it is obvious that the normalized apparent rate constant *κ*_nor_ of the sample in this work is much higher than that of some reported catalysts in literature [47–58], such as core-shell Ag@Pt (0.92 min^−1^g^−1^L), AgNPs/GR-G3.0PAMAM (0.78 min^−1^g^−1^L), rGO/Fe_3_O_4_/Au (0.52 min^−1^g^−1^L). Moreover, for the fifth and tenth cycles, the calculated *κ*_nor_ (0.64 and 0.54 min^−1^g^−1^L) are even higher than these catalysts [51–58]. All these results illustrate that the prepared SnO_2_/Ag microsphere can be taken as a potential efficient catalyst for the reduction of 4-NP.

**Table 2:** Comparison of normalized rate (*κ*_nor_) of different catalysts for the reduction of 4-NP (at room temperature)

Catalyst	*κ*_app_ (min^−1^)	*κ*_nor_ (min^−1^g^−1^L)	Ref.	Issue
Au/TiO_2_	0.17	0.34	[47]	J Catal
Core-shell Ag@Pt	0.15	0.92	[48]	J Catal
Ag/KCC-1	0.6	9	[49]	J Catal
Au/PMMA	0.43	3.25	[50]	J Mol Catal A-Chem
meso-Co_3_O_4_	~0.018	0.23	[51]	Appl. Catal. B: Environ
Co@SiO_2_	0.82	4.08	[52]	Inorg. Chem.
rGO/Fe_3_O_4_/Au	0.69	0.52	[53]	Phys. Chem. Chem. Phys
AgNPs/GR-G3.0PAMAM	1.30	0.78	[54]	J Mol Catal A-Chem
Ag	0.3	1.30	[55]	J. Phys. Chem. C
Ag	0.20	0.29	[56]	Catal Sci Technol
Ag	0.13	0.007	[57]	J Alloy Compd
SnO_2_	0.05	0.04	[58]	Mater. Lett.
SnO_2_/Ag (36 h)—1st	3.10	6.20	This work	**–**
SnO_2_/Ag (36 h)—5th	0.32	0.64	This work	**–**
SnO_2_/Ag (36 h)—10th	0.28	0.54	This work	**–**
Pure SnO_2_	1.24	2.48	This work	**–**
Pure Ag	1.16	2.32	This work	**–**

**Fig. 9:**
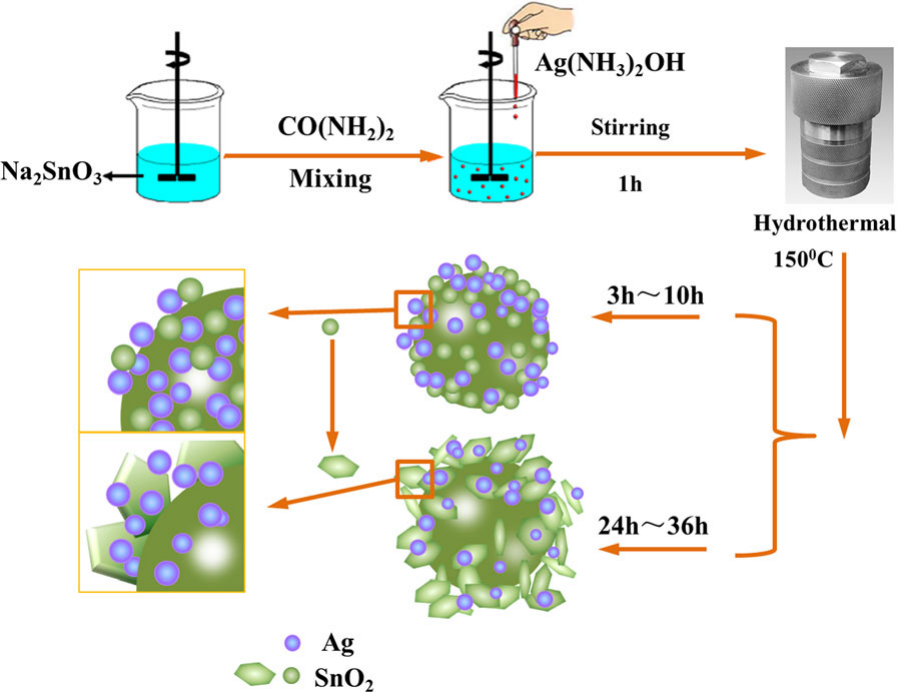
Schematic illustrations of the synthesis of Ag-decorated SnO_2_ microsphere

**Fig. 10:**
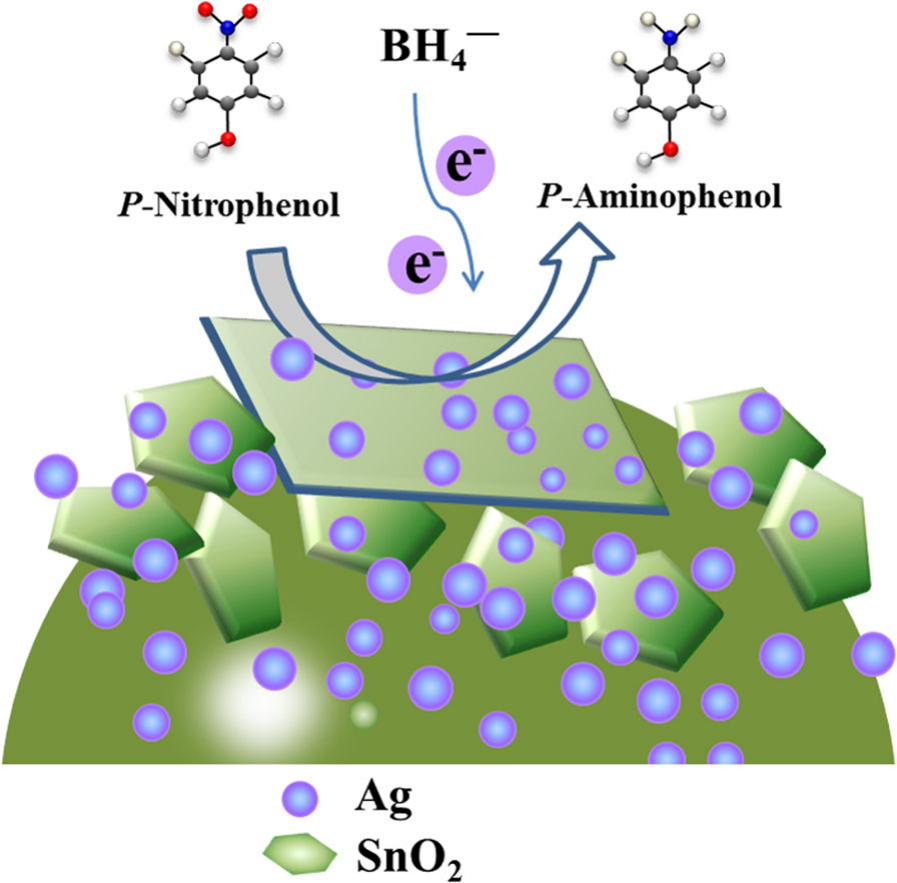
Schematic illustrations of the catalytic reduction of 4-NP to 4-AP over Ag-decorated SnO_2_ microsphere

Based on the previous results and the traditional theory about the catalytic reduction of p-NP by noble metals, the formation mechanism and the origin of the excellent catalytic efficiency of hierarchal Ag-decorated SnO_2_ microsphere were speculated and the schematic is shown in Figs. 9 and 10. In the facile one-pot hydrothermal method, the Ag and SnO_2_ NPs were formed simultaneously in the solution and the freshly born surfaces are inclined to bond with each other. With the increase in hydrothermal time, the SnO_2_ nanoparticles assembled into nanosheets [59] and Ag nanoparticles dispersed in the microsphere. During the catalytic reduction, the Ag nanoparticles start the catalytic reduction by relaying electrons from the donor BH_4_^−^ to the acceptor 4-NP on the adsorption sites of the samples, which was accelerated by the intimate bond between SnO_2_ and Ag NP. Moreover, the dispersed Ag NPs in the microsphere can avoid agglomeration during the catalytic reaction owing to the steric hindrance effect. Furthermore, the synergistic effect of Ag NPs and SnO_2_ nanosheets co-contribute to the excellent catalytic activity of Ag-decorated SnO_2_ composites. In order to verify the assumption, pure SnO_2_ and Ag NPs were synthesized by the similar procedures without the addition of AgNO_3_ and Na_2_SnO_3_, respectively, and then served for the catalytic reduction of 4-NP. The time-dependent UV–Vis spectra and corresponding plots of ln(*C*_*t*_/*C*_0_) versus time for SnO_2_ and Ag NPs are shown in Additional file 1: Figure S8 and Figure S9. It can be observed the reduction also follows a first-order reaction law. The rate constant (*κ*_app_) values calculated from the slope of the linear region were found to be 1.24 min^−1^, and 1.16 min^−1^ for SnO_2_ and Ag, which is lower than that of SnO_2_/Ag. So the excellent catalytic activity of SnO_2_/Ag may arise from the synergistic effect between Ag nanoparticles and SnO_2_ nanosheets. However, the accurate mechanism needs to be further explored.

## Conclusions

In conclusion, hierarchal Ag-decorated SnO_2_ microsphere with uniform Ag nanoparticles and SnO_2_ nanosheets has been successfully prepared by a facile one-pot method. The catalysts prepared by this simple but effective method exhibit excellent catalytic performance for the reduction of 4-NP to 4-AP with *κ*_nor_ of 6.20 min^−1^g^−1^L. Furthermore, the catalyst can sustain high catalytic performance after the first five cycles and could be expected to act as high-efficiency catalysts for the reduction of 4-NP. Moreover, we believe this method can be used as a new strategy to prepare other metal particle-modified semiconductor composites.

## Additional file

Additional file 1: Figure S1. XRD patterns of the SnO_2_/Ag prepared at different temperatures and different contents of mole ratio of Ag. **Figure S2.** Time-dependent UV–Vis spectra for first to fifth cycles for the sample with 5 h hydrothermal time. **Figure S3.** Time-dependent UV–Vis spectra for first to fifth cycles for the sample with 10-h hydrothermal time. **Figure S4.** Time-dependent UV–Vis spectra for first to fifth cycles for the sample with 24-h hydrothermal time. **Figure S5.** Time-dependent UV–Vis spectra for first to fifth cycles for the sample with 36-h hydrothermal time. **Figure S6.** Time-dependent UV–Vis spectra for sixth to tenth cycles for the sample with 36-h hydrothermal time. **Figure S7.** FTIR spectrum of SnO_2_/Ag microsphere after different catalytic cycles. **Figure S8.** Time-dependent UV–Vis spectra for pure SnO_2_ and Ag NPs. **Figure S9.** Plot of ln(*C*_*t*_/*C*_0_) versus reaction time of the pure SnO_2_ and Ag. (DOCX 2159 kb)
