# Supplementary material for: One-Pot Green Synthesis of Ag-Decorated SnO2 Microsphere: an Efficient and Reusable Catalyst for Reduction of 4-Nitrophenol
Source: Nanoscale Res Lett. 2017 Jun 30;12:435. doi: 10.1186/s11671-017-2204-8 (PMC5493606; doi:10.1186/s11671-017-2204-8)
Supplement: Additional file 1: Figure S1. — XRD patterns of the SnO2/Ag prepared at different temperatures and different contents of mole ratio of Ag. Figure S2. Time-dependent UV–Vis spectra for first to fifth cycles for the sample with 5 h hydrothermal time. Figure S3. Time-dependent UV–Vis spectra for first to fifth cycles for the sample with 10-h hydrothermal time. Figure S4. Time-dependent UV–Vis spectra for first to fifth cycles for the sample with 24-h hydrothermal time. Figure S5. Time-dependent UV–Vis spectra for first to fifth cycles for the sample with 36-h hydrothermal time. Figure S6. Time-dependent UV–Vis spectra for sixth to tenth cycles for the sample with 36-h hydrothermal time. Figure S7. FTIR spectrum of SnO2/Ag microsphere after different catalytic cycles. Figure S8. Time-dependent UV–Vis spectra for pure SnO2 and Ag NPs. Figure S9. Plot of ln(C t/C 0) versus reaction time of the pure SnO2 and Ag. (DOCX 2159 kb) [file 11671_2017_2204_MOESM1_ESM.docx]

***Additional file 1***

**One-pot Green Synthesis of Ag Decorated SnO_2_ Microsphere: An Efficient and Reusable Catalyst for Reduction of 4-nitrophenol**

*Hu Min^1^, Zhang Zhenwei ^1^, Luo Chenkun ^1^, Qiao Xiuqing ^1^*

*^1^College of Materials and Chemical Engineering, Hubei Provincial Collaborative Innovation Center for New Energy Microgrid, Key Laboratory of Inorganic Nonmetallic Crystalline and Energy Conversion Materials, China Three Gorges University, Yichang, 443002, Hubei, P. R. China.*

**Nanoscale Research Letters**

* Corresponding authors

E-mail address: qiaoxiuqing@126.com , Tel./Fax: +86-717-6397571

**Figure S1** XRD patterns of the SnO_2_/Ag prepared at different temperatures and different contents of mole ratio of Ag to SnO_2_

**Figure S2** Time dependent UV-Vis spectra for 1^th^-5^th^ cycles for the sample with 5 h hydrothermal time

**Figure S3** Time dependent UV-Vis spectra for 1^th^-5^th^ cycles for the sample with 10 h hydrothermal time

**Figure S4** Time dependent UV-Vis spectra for 1^th^-5^th^ cycles for the sample with 24 h hydrothermal time

**Figure S5** Time dependent UV-Vis spectra for 1^th^-5^th^ cycles for the sample with 36 h hydrothermal time

**Figure S6** Time dependent UV-Vis spectra for 6^th^-10^th^ cycles for the sample with 36 h hydrothermal time

**Figure S7** FTIR spectrum of SnO_2_/Ag microsphere after different catalytic cycles

**Figure S8** Time dependent UV-Vis spectra for pure SnO_2_ and Ag NPs

**Figure S9** Plot of ln(C_t_/C_0_) versus reaction time of the pure SnO_2_ and Ag
